# Pathway centrality in protein interaction networks identifies putative functional mediating pathways in pulmonary disease

**DOI:** 10.1038/s41598-019-42299-3

**Published:** 2019-04-10

**Authors:** Jisoo Park, Benjamin J. Hescott, Donna K. Slonim

**Affiliations:** 1 School of Medicine, University of California, San Diego, CA 92093 USA; 2 College of Computer and Information Science, Northeastern University, Boston, MA 02115 USA; 3 Department of Computer Science, Tufts University, Medford, MA 02155 USA; 4 Department of Immunology, Tufts University School of Medicine, Boston, MA 02111 USA

## Abstract

Identification of functional pathways mediating molecular responses may lead to better understanding of disease processes and suggest new therapeutic approaches. We introduce a method to detect such mediating functions using topological properties of protein-protein interaction networks. We define the concept of pathway centrality, a measure of communication between disease genes and differentially expressed genes. Using pathway centrality, we identify mediating pathways in three pulmonary diseases (asthma; bronchopulmonary dysplasia (BPD); and chronic obstructive pulmonary disease (COPD)). We systematically evaluate the significance of all identified central pathways using genetic interactions. Mediating pathways shared by all three pulmonary disorders favor innate immune and inflammation-related processes, including toll-like receptor (TLR) signaling, PDGF- and angiotensin-regulated airway remodeling, the JAK-STAT signaling pathway, and interferon gamma. Disease-specific mediators, such as neurodevelopmental processes in BPD or adhesion molecules in COPD, are also highlighted. Some of our findings implicate pathways already in development as drug targets, while others may suggest new therapeutic approaches.

## Introduction

Identification of biological mechanisms underlying disease is crucial to the development of new therapeutic strategies. A common approach to understanding disease processes is through functional analysis of either genes that are differentially expressed in disease, or of putative disease-causing genes identified through GWAS or targeted studies. However, it has long been noted that causal disease genes are not necessarily differentially expressed, and that differential expression does not easily lead to the discovery of disease genes^1^. In many cases, differential expression predominantly reflects the tissue-specific downstream effects of a disease-causing process that integrates complex genetic and environmental responses. This makes differentially expressed genes useful as diagnostic markers, but often poor as therapeutic targets^2^. Conversely, functional analysis of causal disease genes often doesn’t fully explain how these downstream responses occur^3^. To bridge this gap, there is a need to identify the functional pathways mediating the transcriptional response in disease.

We do so by considering the roles that proteins corresponding to disease genes or differentially expressed genes play in protein-protein interaction networks. Such networks are often represented by graphs, where the nodes represent individual proteins and edges represent physical interactions between pairs of proteins. We postulate that the functional pathways mediating disease response will disproportionately reflect communication between these two sets of proteins, and that if we look for such “central” pathways we will find mediators. This topological property is well captured by the concept of betweenness centrality, where *fractional betweenness* characterizes how many of the shortest paths between all pairs of nodes passes through a given node^4,5^. We start by introducing a variant of node betweenness called *disease fractional betweenness*, which counts only the shortest paths between disease genes and differentially expressed genes for a given node. To identify putative mediating pathways in disease response, we then introduce a generalized notion of group centrality^6^ called *pathway centrality*, which aggregates disease fractional betweenness scores across all genes in a given pathway.

We apply our novel pathway-centrality method to three pulmonary diseases that primarily affect patients at different stages of life: bronchopulmonary dysplasia (BPD), a neonatal complication of preterm birth; asthma, which is relevant across the lifespan but is often diagnosed in childhood; and chronic obstructive pulmonary disease (COPD), which encompasses a number of progressive lung disorders that predominantly affect the elderly. For each disease, we examine the pathway centrality of all KEGG pathways and all Biological Process gene sets from the Gene Ontology. Approximating significance via permutation, we identify sets with significantly high pathway centrality as functional mediating pathways. We use a separate collection of genetic interaction data to demonstrate systematically that the identified pathways fill the desired topological roles in the network. Our approach finds candidate mediators not discovered through traditional hypergeometric enrichment of the disease gene or differentially expressed gene sets. We also discuss published evidence consistent with our disease-specific findings.

While group centrality has previously been applied via an optimization framework to discover new groups of important nodes in gene networks^7^, it has not yet been used to identify the functional gene sets playing a pivotal role in disease. This approach is the first to identify functional pathways mediating signals between disease genes and differentially expressed genes by measuring disease-specific communication passing through pathway genes in protein-protein interaction networks. We emphasize that this aim is different from that of finding disease genes themselves. The pathways we identify appear to mediate cellular response to disease states, and yet their component genes may be neither mutated nor significantly differentially expressed in disease.

The prior work most relevant to this effort is a collection of related results linking expression quantitative trait loci (eQTLs) to differentially expressed genes via protein-protein, protein-DNA, and phosphorylation networks. These studies were initially intended to find the causal gene in a disease-linked locus by tracing back the path of information flow from selected target genes that are differentially expressed^8–11^. Such efforts are related to ours in the sense that they examine information flow between genes linked to disease and differentially expressed genes. However, these analyses focus on only selected differentially expressed genes, and they do not directly point to mediating biological functions. Our focus is on the disease-related pathways, and our aim is to identify underlying biological functions that mediate cellular response in disease, rather than to identify genes with disease-causing mutations or variants.

We find that pathways involved in innate immunity, and several related signaling pathways including PDGF, JAK/STAT, and toll-like receptor signaling, are common mediators of all three pulmonary disorders. Disease-specific mediators include lipid homeostasis pathways in COPD, integrin mediated cell adhesion in asthma, and insulin-like growth factor receptor signaling in BPD. While a number of our findings have already been proposed as disease mediating pathways, diagnostic tools, or sources of therapeutic targets in previous publications, we discovered several novel mediators that may suggest new therapeutic approaches for these diseases.

## Results

### A pathway centrality approach to finding mediating pathways in disease

To discover functional processes mediating disease response, we start by identifying a set of disease genes whose mutations or variants have been shown to cause the indicated disease. Additionally, we identify differentially expressed genes from previously-published transcriptomic profiles of disease-relevant tissues. Our protein-protein interaction network is derived from the HIPPIE database^12^. Details describing network construction, sources of disease genes, and expression data appear in the Methods section.

We define pathway centrality to measure the amount of information a set of pathway genes handles by counting the shortest paths linking disease genes and differentially expressed genes. Specifically, let *V* be the set of all vertices in a protein-protein interaction network. While the classical definition of fractional betweenness (FB) for a node *v* ∈ *V* is the fraction of shortest paths between all pairs of nodes in the network passing through *v*, our pathway centrality score is based on a modified FB score which we call disease fractional betweenness (DFB). For node *v* and disease *d*, disease fractional betweenness only reflects the shortest paths between disease genes and differentially expressed genes that pass through *v*.

Formally, if *D*(*d*) is the set of genes in *V* associated with disease *d*, *E*(*d*) is the set of differentially expressed genes in *V* for disease *d*, *B*_s,t_ is the number of shortest paths between s and t, and *B*_s,t_(v) is the number of shortest paths between *s* and *t* that pass through *v*, then disease fractional betweenness is defined as:

1 $$DFB(v)={\sum }_{s\in D(d)\backslash \{v\},t\in E(d)\backslash \{v\}}\frac{{B}_{s,t}(v)}{{B}_{s,t}}$$

We then define pathway centrality (PC) as the average disease fractional betweenness score across all genes in a pathway. Specifically, for a pathway *k* containing the gene set *P*(*k*), pathway centrality *PC*(*k*) is defined as:

2 $$PC(k)=\frac{{\sum }_{v\in P(k)}DFB(v)}{|P(k)|}$$

Once we have computed the pathway centrality score for a pathway *k*, we need a method to assess significance by characterizing how surprising it is to see a score at least as large as *PC*(*k*). The significance of the observed *PC*(*k*) score is assessed using a null distribution derived by selecting 10,000 random gene sets of size |*P*(*k*)|. The observed fraction of random sets with higher pathway centrality scores than *PC*(*k*) is reported as p_cent_(*k*), a rough measure of significance. (When the gene set or pathway is clear from context, we omit the argument and just write p_cent_).

However, developing a valid null distribution requires a non-trivial strategy for random gene set sampling, as pathway genes are known *a priori* to be relatively central in protein interaction networks. Thus, it is likely that pathway genes have higher fractional betweenness than those that are not involved in well-annotated functional processes. Another fundamental issue is that different centrality measurements are highly correlated^13^; in particular, fractional betweenness centrality is correlated with degree centrality. This can be problematic because the degree of a gene strongly correlates with how “popular” or well-annotated the gene is. Thus, our pathway centrality analysis could favor pathways containing well-studied genes unless we force the random sampling process to mimic the original degree distribution.

To overcome such biases, we therefore impose restrictions on our random gene set sampling process. First, we require our random samples to be drawn from the collection of genes belonging to at least one pathway. We also want the degree distribution of each random gene set to resemble that of the candidate mediating pathway. However, because there are only a few high-degree genes, we cannot necessarily exactly match the degree of high degree genes in the candidate pathway while choosing from a sufficiently large population of alternatives. Thus, we “bin” the nodes by degree, such that similarly high-degree nodes are placed into a single bin for sampling. This process, described further in the Methods section, ensures that there are a sufficient number of choices for approximately matching the degree of high-degree nodes in mediating pathways.

### Finding disease-specific and shared mediators of pulmonary disease

Pathways with significant pathway centrality in individual pulmonary data sets include known disease mediators, along with other pathways whose disease-relevance has not yet been identified. Tables 1 and 2 show a selection of the top Gene Ontology Biological Processes (GO BP) terms and KEGG pathways, respectively, identified in exactly one of the pulmonary disorders; the featured terms were manually selected to represent the functional range of the significant results. There are fewer significant KEGG pathways, but they tend to implicate similar functions to those found using GO BP gene sets. Full results for both pathway collections are available in Supplementary Table S1.

**Table 1:** Disease-specific mediating GO Biological Processes for each pulmonary disorder. Selected GO terms with p_cent_ < 0.01 (highlighted in bold) in exactly one of the pulmonary disorders. Full results are available in Supplementary Table S1. Colors indicate functional classes; immune: blue; oxygen/oxidative-stress response: green; signaling: yellow; neurodevelopment: orange; adhesion/ECM/structural: red; metabolic: pink; homeostasis: purple. An asterisk on the right (“Not HG”) means that neither that pathway nor any similar pathway is significantly enriched (with hypergeometric (HG) FDR < 0.05) in either the disease gene set or the differentially-expressed gene set for the indicated disease.

	Pathway	asthma p-value	BPD p-value	COPD p-value	Not HG
blue	Positive Regulation of Mast Cell Activation	**0.0013**	0.0651	0.2274	
blue	Myeloid Cell Activation Involved in Immune Response	**0.0016**	0.1200	0.2881	
blue	Leukocyte Degranulation	**0.0075**	0.1483	0.3737	
blue	T Cell Mediated Immunity	**0.0033**	0.0613	0.9692	
blue	Antigen Processing… via MHC class II	**0.0050**	0.0560	0.6590	
blue	Positive Regulation of B Cell Differentiation	**0.0068**	0.0942	0.2204	*
blue	Macrophage Activation Involved in Immune Response	**0.0077**	0.0705	0.1990	
green	Cation Transport	**0.0004**	0.1773	0.1186	
green	Positive Regulation of Calcium Mediated Signaling	**0.0052**	0.0541	0.4756	
green	Positive Regulation of Nitric Oxide Synthase Activity	**0.0053**	0.1376	0.1330	
yellow	Positive Regulation of ERBB Signaling Pathway	**0.0030**	0.1172	0.2060	*
red	Positive Regulation of Cell Adhesion Mediated by Integrin	**0.0080**	0.0743	0.3015	
pink	Amyloid Precursor Protein Metabolic Process	**0.0027**	0.8259	0.7919	*
pink	Regulation of Glucose Import	**0.0044**	0.0944	0.2334	
blue	Negative Regulation of TNF Mediated Signaling Pathway	0.3954	**0.0042**	0.1453	
blue	Negative Regulation of Antigen Receptor Mediated Signaling	0.2772	**0.0004**	0.2115	*
blue	Negative Regulation of T Cell Receptor Signaling Pathway	0.2701	**0.0005**	0.2132	*
blue	B Cell Mediated Immunity	0.1489	**0.0006**	0.0667	*
blue	Regulation of Interleukin 1 Secretion	0.4228	**0.0083**	0.4387	
yellow	Epidermal Growth Factor Receptor Signaling Pathway	0.1158	**0.0000**	0.1275	*
yellow	Regulation of Insulin Like Growth Factor Receptor Signaling	0.4711	**0.0002**	0.1137	*
yellow	Activation of MAPKK Activity	0.1732	**0.0033**	0.4494	*
yellow	VEGF Receptor Signaling Pathway	0.1577	**0.0063**	0.0732	*
orange	Forebrain Development	0.3310	**0.0000**	0.1579	*
orange	Cerebral Cortex Cell Migration	0.3632	**0.0016**	0.9484	*
orange	Neuroepithelial Cell Differentiation	0.0947	**0.0031**	0.9680	*
orange	Auditory Receptor Cell Differentiation	0.0814	**0.0008**	0.8433	*
red	Positive Reg. of Substrate Adhesion Dependent Cell Spreading	0.4722	**0.0005**	0.0626	*
red	Actin Filament Organization	0.1692	**0.0034**	0.1645	
red	Wound Healing	0.0665	**0.0002**	0.1049	
pink	Regulation of Glucose Metabolic Process	0.5563	**0.0041**	0.2729	*
pink	Positive Regulation of Phospholipid Metabolic Process	0.1055	**0.0006**	0.1371	
blue	Negative Regulation of TGF Beta Receptor Signaling Pathway	0.1454	0.1127	**0.0001**	
blue	Positive Regulation of Adaptive Immune Response	0.2672	0.2108	**0.0004**	
blue	Positive Regulation of Natural Killer Cell Activation	0.3802	0.1776	**0.0026**	*
blue	Negative Regulation of Leukocyte Migration	0.1067	0.2482	**0.0038**	
green	Response to Metal Ion	0.0588	0.0729	**0.0000**	
green	Reactive Oxygen Species Metabolic Process	0.1188	0.1878	**0.0003**	
green	Response to Increased Oxygen Levels	0.6638	0.2764	**0.0034**	
green	Negative Regulation of Apoptotic Signaling Pathway	0.3339	0.1617	**0.0023**	
red	Regulation of Extracellular Matrix Organization	0.1161	0.0692	**0.0006**	
pink	Regulation of Amyloid Precursor Protein Catabolic Process	0.1834	0.1097	**0.0038**	*
violet	Lipid Homeostasis	0.5030	0.1191	**0.0000**	*
violet	Anion Homeostasis	0.3261	0.0796	**0.0022**	
violet	Acylglycerol Homeostasis	0.6552	0.1044	**0.0033**	*

**Table 2:** Disease-specific mediating KEGG pathways for each pulmonary disorder. Selected pathways with p_cent_ < 0.05 (highlighted in bold) in exactly one of the pulmonary disorders. The color code indicating functional classes is the same as in as Table 1.

	Pathway	asthma p-value	BPD p-value	COPD p-value	Not HG
blue	FC Gamma R Mediated Phagocytosis	**0.0081**	0.1145	0.1830	
blue	B Cell Receptor Signaling Pathway	**0.0133**	0.1030	0.1444	
blue	Endocytosis	**0.0092**	0.0523	0.4102	
yellow	Neurotrophin Signaling Pathway	**0.0464**	0.0921	0.4021	*
pink	Glutathione Metabolism	**0.0265**	0.8872	0.2423	
pink	Sulfur Metabolism	**0.0049**	1.0000	0.5412	*
blue	Allograft Rejection	0.3217	**0.0003**	0.1082	
blue	Intestinal Immune Network for IGA production	0.3588	**0.0004**	0.2877	
blue	Epithelial Cell Signaling in Helicobacter Pylori Infection	0.2658	**0.0060**	0.2032	
blue	Graft Versus Host Disease	0.3037	**0.0019**	0.2215	
green	Calcium Signaling Pathway	0.6121	**0.0331**	0.4865	*
yellow	GNRH Signaling Pathway	0.5226	**0.0021**	0.1937	*
yellow	MAPK Signaling Pathway	0.1084	**0.0038**	0.2018	
yellow	ERBB Signaling Pathway	0.2109	**0.0122**	0.0834	*
yellow	VEGF Signaling Pathway	0.2003	**0.0153**	0.1221	*
orange	Dorso-ventral Axis Formation	0.4219	**0.0112**	0.2261	*
red	Regulation of Actin Cytoskeleton	0.3866	**0.0000**	0.0684	
red	Tight Junction	0.5570	**0.0237**	0.6362	*
red	Gap Junction	0.4007	**0.0022**	0.1372	
red	Cell Adhesion Molecules CAMS	0.2227	0.0884	**0.0014**	
pink	Arachidonic Acid Metabolism	0.1433	0.8886	**0.0048**	
pink	Arginine and Proline Metabolism	0.5466	0.4317	**0.0466**	*

The functional annotations in Table 1 predictably identify immune processes as mediators of each of the three pulmonary disorders, but they highlight different aspects of immunity and signaling that appear to distinguish the individual diseases. Significant immune mediators in asthma include cells of both myeloid and lymphoid origin, emphasizing the role of the innate immune response (e.g., mast cell activation; macrophage activation) in the disease. In contrast, those unique to COPD highlight adaptive immunity. Because both innate and adaptive immune pathways are known to play a role in both diseases^14^, these results suggest that the innate immune pathways mediating the COPD response (such as Th1) are also implicated in other airway disorders.

**Figure 1:**
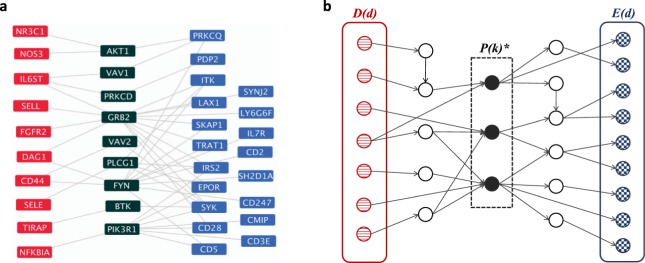
(**a**) Mediating topology of the JAK-STAT signaling pathway in BPD. One of the significant mediating pathways for bronchopulmonary dysplasia (BPD) is the KEGG JAK-STAT signaling pathway. Here, mediating pathway genes are colored dark green and appear between BPD disease genes (red) and differentially expressed genes (blue). Edges directly linking the disease genes to the pathway or the pathway to the differentially expressed genes in BPD are shown. The pathway is visualized using cytoscape^49^. **(b)** Idealized topological property of disease-mediating pathways. Let *D*(*d*) be a set of disease genes and *E*(*d*) be a set of differentially expressed genes for disease *d*. Genes in *P*(*k*)*, a significant mediating pathway of disease *d* with *p*_*cent*_(*k*) < 0.05, are expected to play central roles in passing signals from *D*(*d*) to *E*(*d*).

When we look at pathways that play a significant role across all three pulmonary disorders (Table 3), we again find a preponderance of inflammatory and immune processes. Significant immune pathways across all three data sets largely focus on innate immunity, though there are some predominantly adaptive processes (e.g. T cell signaling) and others (IL-1, cytokine signaling) that have roles in both. Several specific signaling pathways and systems are implicated, including JAK/STAT signaling, toll-like receptor signaling, PDGF, interferon gamma, and the renin-angiotensin and complement systems. (Fig. 1(a) shows the JAK/STAT pathway topology in BPD as an example).

**Table 3:** Common mediating pathways of all three pulmonary disorders. Selected GO Biological Process terms and KEGG pathways with significant (p_cent_ < 0.05) in all three pulmonary disorders. The color key is as in Table 1. Full results are available in Supplementary Table S1.

	Pathway	asthma p-value	BPD p-value	COPD p-value
**GO biological process terms**				
blue	Innate Immune Response	0.0000	0.0000	0.0000
blue	Response to Bacterium	0.0001	0.0001	0.0000
blue	Cellular Response to Cytokine Stimulus	0.0001	0.0002	0.0000
blue	Cellular Response to Interferon Gamma	0.0005	0.0008	0.0047
blue	Positive Regulation of Alpha Beta T Cell Activation	0.0072	0.0011	0.0012
blue	Response to Interleukin 1	0.0117	0.0045	0.0055
blue	Regulation of Toll Like Receptor Signaling Pathway	0.0100	0.0266	0.0039
yellow	Regulation of PDGF Receptor Signaling	0.0054	0.0001	0.0011
red	Platelet Degranulation	0.0000	0.0000	0.0000
red	Regulation of Homotypic Cell Cell Adhesion	0.0010	0.0000	0.0002
red	Regulation of Vasoconstriction	0.0003	0.0044	0.0000
pink	Regulation of Lipid Metabolic Process	0.0005	0.0072	0.0005
**KEGG pathways**				
blue	Cytokine Cytokine Receptor Interaction	0.0000	0.0000	0.0000
blue	Chemokine Signaling Pathway	0.0029	0.0022	0.0242
blue	Complement and Coagulation Cascades	0.0117	0.0000	0.0166
blue	Natural Killer Cell Mediated Cytotoxicity	0.0165	0.0029	0.0243
yellow	JAK-STAT Signaling Pathway	0.0000	0.0091	0.0137
red	Renin Angiotensin System	0.0095	0.0007	0.0025

To distinguish our significant mediating pathways from enriched functions within the disease-related genes, we next assessed whether the identified pathways were significantly enriched (hypergeometric, Benjamini-Hochberg adjusted FDR < 0.05) in either the disease gene set or the differentially-expressed gene set for the indicated disease. Many significant mediating pathways are not detectable by functional enrichment analysis. Pathways in Tables 1 and 2 marked with an asterisk in the right-most column meet a more conservative criterion: no significant enrichment of either the indicated pathways, nor any with a substantially similar function, was detected. For example, in BPD, the GO gene set “Epidermal Growth Factor Receptor Signaling Pathway” is identified as a significant mediator. Yet the disease and differentially expressed genes in BPD did not show significant enrichment of this or any gene sets related to EGFR. The ability of pathway centrality to identify such mediating gene sets suggests that the use of network structure in computing pathway centrality implicates pathways that would not be found by traditional enrichment approaches. Supplementary Table S2 lists the hypergeometric enrichment scores for disease and differentially expressed gene sets for all significant pathways.

### Genetic interaction data confirms the mediating topology of identified pathways

One way to verify that the proposed mediating pathways truly include genes mediating responses to disease genes would be to identify an excess of epistatic relationships between them. For example, if a mediating pathway looks like that shown in Fig. 1(b), one might expect a higher likelihood of seeing certain kinds of genetic interactions between a disease gene *g*_*D*_ in set *D* and a mediating gene *g*_*P*_ from set *P*(*k*)* than between *g*_*D*_ and genes that are not in a mediating pathway for that disease. The genetic interactions of most interest are “alleviating” or positive interactions, where the deleterious effect of the double mutant of both *g*_*D*_ and *g*_*P*_ is less severe than would be predicted by combining the independent effects of individual mutations in *g*_*D*_ or *g*_*P*_. Such relationships might arise when *g*_*P*_ is part of a pathway mediating the response to *g*_*D*_.

However, because it is difficult to find sufficient numbers of verified human genetic interactions, we additionally collected alleviating (positive) genetic and phenotypic suppression genetic interactions from the model organisms *Schizosaccharomyces pombe, Saccharomyces cerevisiae, Drosophila melanogaster*, and *Caenorhabditis elegans*. For each gene set *P*(*k*), we then define p_med_(*k*), the probability of finding the observed number of positive genetic interactions between disease genes and genes in *P*(*k*) through a similar binning approach to that used for *p*_cent_(*k*).

**Figure 2:**
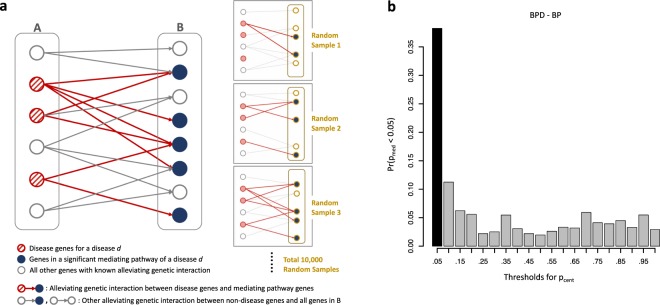
(**a**) Systematic confirmation of significant mediating pathways. If identified pathways are truly mediating a disease response, the pathway genes are likely to be downstream of the corresponding disease genes. This topology can be captured by an excess of epistatic relationships between disease genes and a mediating pathway genes. To test whether our significant disease-mediating pathway genes form such topologies with the disease genes more than others, we first collected alleviating genetic interactions from human and a few model organisms. A is the set of all disease genes and B is the set of “downstream” genes that have alleviating genetic interactions with genes in A. We count *x*, the number of alleviating genetic interactions between disease genes and genes belonging to the specific identified mediating pathway (here *x* is the number of red edges), and then assess significance by calculating the probability that a random gene set of equal size has at least *x* alleviating genetic interactions with the disease genes. The null distribution is learned from 10,000 random samples drawn from a pool of genes of any known alleviating genetic interactions (set B). (**b**) Histogram showing the relationship between p_cent_ and p_med_, for GO BP gene sets and BPD. Each bar represents the fraction of pathways with p_cent_ <0.05 in the designated range (from the labeled value minus 0.05, to the labeled value) that also have p_med_ <0.05. The plot shows that many of pathways with significant *p*_cent_ scores (below 0.05, shown by the dark bar) also have low *p*_med_ scores.

To assess how surprising it is to see the observed number of such positive genetic interactions between the disease genes and the pathway, we compute a null distribution of the number of alleviating or suppressing interactions between the same set of disease genes and 10,000 random gene sets of the same size as the candidate mediating pathway. Again, we impose restrictions on the source of our random gene sets: they must be drawn from a pool of genes that belong to at least one pathway in the collection and that are downstream genes of any alleviating genetic or phenotypic suppression interactions (Fig. 2(a)). We used a binning strategy analogous to that for *p*_cent_ to approximately match the in-degree distributions of known downstream genes of disease genes and our random samples.

We would then like to assess whether a significant disease-mediating pathway *k* having a low p_cent_ value is more likely to have a low p_med_ value. If it does, that indicates an excess of positive genetic interactions between disease genes and genes in *P*(*k*), suggesting that the disease genes are potentially located “upstream” of the mediating pathway.

To capture this relationship, we compute a histogram of the percentage of p_med_ scores below 0.05 for each of 20 quantiles of possible p_cent_ values. A sample plot of these frequencies is shown in Fig. 2(b); plots for all data sets and gene set collections are available as Supplementary Figure S1. One-sided, non-parametric Wilcoxon tests comparing the distribution of p_med_ values in the first quantile (i.e., p_cent_ below 0.05) to the distribution in all the other quantiles (p_cent_ ≥ 0.05) confirm that the most significant mediating pathways are associated with lower p_med_ scores in all the pulmonary disease cases. These (raw) significance values are shown in Table 4.

**Table 4:** Significance of relationships between p_cent_ and p_med_. The table shows the resulting p-values of one-sided Wilcoxon tests comparing the distribution of *p*_med_ values of the pathways with *p*_cent_ < 0.05 to that of the pathways with *p*_cent_ ≥ 0.05. *p*_med_ values of the pathways with *p*_cent_ < 0.05 are significantly smaller than those of all other pathways.

	Wilcoxon p-value		
	Asthma	BPD	COPD
BP	2.57E-05	5.78E-26	0.0124
KEGG	0.0263	2.18E-07	0.0106

As an example, the KEGG MAPK signaling pathway has a p_cent_ score of 0.0038 in BPD. There are ten supporting alleviating genetic interactions between BPD disease genes and genes in the KEGG MAPK signaling pathway: PLAU-HRAS, PLAU-MAP2K1, TIRAP-MP3K7, TIRAP-TRAF6, TLR4-ECSIT, TNF-CHUK, TNF-RAC, FGFR2-RAC1, FGFR2-KRAS, and FGFR2-CDC42; leading to a corresponding p_med_ score below 0.0001. This pathway is one of the more than a third of pathways with p_cent_ below 0.05 in BPD whose p_med_ score is also below 0.05, contributing to the dark bar on the left-hand side of Fig. 2(b).

Both the Wilcoxon tests and the plots support the conclusion that there is enrichment of alleviating genetic relationships between disease genes and pathway genes for the pathways whose p_cent_ values are deemed significant, confirming that pathway centrality is indeed generally finding gene sets with the desired network topology.

## Discussion

We have introduced a new centrality-based method to identify functional pathways mediating disease responses by dominating communication between disease and differentially expressed genes. Although there are many issues with the available genetic interaction data, the conservation of genetic interactions across species being one of the most salient, systematic evaluation using genetic interactions confirms that our method finds pathways with the desired network topology. Recent work discussing the plethora of GWAS hits with modest effects suggests that functional network analysis is essential for translating these hits into actionable knowledge^15^. Our work illustrates one such approach.

The journey from identifying disease-relevant pathways to the discovery of novel and effective therapeutics may involve multiple steps, but it has been widely considered to be a promising approach to drug-discovery in the genomic era. One question is whether the pathway is simply a list of potential targets. In many cases the story will be more complex than that; in particular, it is not always plausible that there is a single targetable molecule that will disrupt the relevant processes^16^. Still, recent work in airway diseases emphasizes that understanding the relevant pathways is essential to discovering more effective treatments modifying their functions^17^.

Several of the pathways identified by pathway centrality are already under consideration as therapeutic targets for the indicated disease. For example, the KEGG gene set “Cell Adhesion Molecules (CAMS)” tops the unique KEGG list in COPD (Supplementary Table S1). Prior work suggests that adhesion molecules also play a significant role in the pathogenesis of COPD^18^, and that clinical trials of therapeutics regulating adhesion and integrin are underway for both COPD and asthma^19^. “Positive regulation of cell adhesion mediated by integrin,” a non-overlapping GO gene set we uniquely identified as a mediator in asthma, has more recently been considered as a targetable process to reduce airway hyper-responsiveness^20^. Therefore, it is important that although adhesion and leukocyte chemotaxis are important to all three disorders^21–23^, the pathway centrality approach highlights different sets of genes mediating these responses.

Similarly, the JAK/STAT pathway, implicated as a mediator in all three disorders, has been suggested as an asthma target through inhibitors of activating cytokines and receptors^24^. JAK pathway inhibitors are in development for a number of inflammatory disorders^25^. Work in animal models has suggested that targeting this pathway can reduce airway hyperresponsiveness but has widespread effects, leading to efforts to develop inhaled therapeutics targeting the JAK pathway for both COPD and asthma^26^. The role of JAK/STAT signaling in bronchopulmonary dysplasia is less clear, but it has been suggested that it plays a role in airway smooth muscle mitogenesis, implicated in both asthma and BPD^27^, and postulated that it may be an alternative mediator of the oxidative stress response in both diseases^28^. (Fig. 1(a) shows a subset of this pathway and the PPI network for BPD.) Thus, our work suggests that if safe and effective compounds targeting this pathway are developed for asthma or COPD, there may be some potential for their relevance in BPD as well.

Toll-like receptor (TLR) signaling, which activates the innate immune response and was implicated in all three diseases, is another familiar part of the story of airway hyperreactivity and fetal lung development^29^. TLR polymorphisms have been linked to an increased risk of developing BPD^30^, and TLR agonists are already being tested for therapeutic efficacy in asthma^31^. However, the role of this system in COPD is not as well studied. Aspects of the innate immune response are often demonstrably suppressed in COPD patients^32^, consistent with our results in the previous section showing that most COPD-specific immune response pathways regulate the adaptive immune response. TLR polymorphisms also play a role in disease susceptibility and severity^33,34^. Our work therefore provides evidence for a role for TLR pathways in the diagnosis, stratification, and treatment of COPD.

Unique to BPD is a collection of mediating neurodevelopmental pathways. BPD has long been known to be associated with worse neurodevelopmental outcomes than those observed in infants without BPD delivered at similar gestational ages^35^. Recently, BPD has been shown to be associated with a measurable decrease in IQ^36^. Whether that decrease is a consequence of BPD or arises from a common cause is unknown. Our results, showing neurodevelopmental pathways mediating the expression response in the blood of 5 day old infants who go on to develop BPD, suggest that some of the association is likely due to molecular causes, rather than, say, the consequences of neonatal hypoxia.

The identification of common mediating pathways in airway disease throughout the lifespan may shed light on potential implications of neonatal or childhood respiratory disorders. While the discovery of common immune pathways here is not surprising, the distinction between which pathways appear to be disease specific and which are common may be informative. Meanwhile, the shared involvement of vasoconstriction and adhesion pathways suggests a different commonality that might be exploited to mitigate later pulmonary issues in children with BPD or asthma.

One potential issue affecting our work is that disease genes and differentially expressed genes are not necessarily distinct gene sets. We therefore treat genes in both sets as differentially expressed genes only. However, we also note that the overlap between these groups is sufficiently small (asthma: 1, BPD: 1, COPD: 9) that it is unlikely to substantially affect our results. Similarly, one could imagine that separating up- and down-regulated differentially-expressed genes might provide further power for this type of analysis. Exploring this hypothesis could be a fruitful avenue for future work, although the lack of directionality in many of the considered gene sets makes implementation of such an approach non-trivial.

Our method also suggests a new way to analyze protein-protein interaction networks in the context of a disease of interest. Disease-specific network analysis usually excludes genes not known to be associated with the disease. Our approach enables disease-specific analysis without altering the topology of protein-protein interaction networks, and accounts for roles of neighboring genes in disease-related communication. It also can be applied to any type of molecular network. We see an opportunity to improve the chance for finding novel disease-mediating pathways by combining networks of protein-protein interactions and other types of molecular data, such as transcription factor-target interactions.

Overall, we have demonstrated that our pathway-centrality method finds functional mediators of disease using complementary interaction data. We have seen that the identified pathways include some therapeutic targets already in development, suggesting that others may be similarly promising but novel. Our findings confirm established connections of pulmonary disorders with inflammatory and immune processes, signaling processes, and airway remodeling. We expect that this approach may be applied more generally to discover relevant and informative pathways for any disease or phenotype of interest.

## Methods

### Protein-protein and genetic interaction networks

We use two biological networks in our experiments. To measure pathway centrality, physical protein-protein interactions were collected from the Human Integrated Protein-Protein Interaction rEference (HIPPIE)^12^ database. HIPPIE contains experimentally verified protein interactions with confidence scores. We downloaded the interaction data (version 2.1) on September 8, 2017 and selected only those interactions described as “high confidence” (≥0.73), as these interactions are supported by more reliable evidence. We worked with the largest connected component extracted from the network, which contains 62,679 interactions between 12,064 proteins. Note that we use the protein to gene mapping provided by the HIPPIE database to map protein-protein interactions. Thus, although our canonical entities in the network are represented by Entrez gene identifiers, we refer to these interactions as protein-protein interactions throughout the manuscript.

To compute p_med_ scores based on genetic interaction data, we looked for genetic interaction data featuring alleviating (positive) genetic and phenotypic suppression interactions. Because relatively few of these genetic interactions are known for humans, we additionally collected such interactions from *Schizosaccharomyces pombe, Saccharomyces cerevisiae, Drosophila melanogaster*, and *Caenorhabditis elegans*. These interactions came from BioGRID^37^ (version 3.4.160), the Saccharomyces Genome Database (SGD project, http://www.yeastgenome.org), and Flybase^38^, all downloaded on May 23, 2018. To find human homologous interaction pairs, we use a mapping downloaded from the HomoloGene database^39^ on July 19, 2016 (the current version was uploaded on April 14, 2014). This approach yielded 9,395 pairs of putative positive human genetic interactions.

### Disease-related genes and functional gene sets

For Asthma and COPD, 111 and 192 disease genes were collected from recent reviews of asthma^40^ and COPD genes^41^. Lacking a similar-scale summary of BPD genetics, we collected 81 genes associated with BPD from Online Mendelian Inheritance in Man (OMIM)^42^ and Genopedia^43^, as described in^44^. These datasets were downloaded on April 4, 2018.

Gene expression microarray profiles for Asthma and BPD were obtained from the GEO database (accession numbers GSE4302 and GSE32472, respectively). The first measured differential expression in airway epithelial cells between healthy controls and asthma patients^45^, while the second examined expression in peripheral blood cells from infants born preterm with or without BPD^46^. From the preterm study, we used only samples taken on postnatal day 5 (the earliest time point). We selected as differentially expressed genes between disease and control groups those with an adjusted Benjamini-Hochberg t-test p-value below 0.01, yielding 82 and 422 expression-related genes in asthma and BPD, respectively. For COPD, we downloaded RNA-seq EdgeR results comparing expression in lung cells from COPD patients and controls (GSE57148)^47^. Relying on the analysis methods from the original study, since RNA-seq and Affymetrix analysis pipelines differ, we identified 266 significantly differentially expressed genes with an EdgeR q-value below 10^−10^.

Note that there may be disease genes that are also differentially expressed in that disease. To avoid confusion about how to use these in computing pathway centrality, we removed genes from the disease gene sets that also appeared in the corresponding set of differentially expressed genes. Disease genes and differentially expressed genes are further excluded from our experiments if they do not have any known interactions with other genes in our protein-protein interaction network data. Removing those genes without known interactions results in 86 (asthma), 70 (BPD) and 146 (COPD) disease genes, and 42 (asthma), 216 (BPD) and 198 (COPD) differentially expressed genes.

Supplementary Figure S2 shows the overlaps between these three disease gene sets and between the three differentially-expressed gene sets. There is very little overlap seen in the differentially-expressed gene sets, and at least half the disease genes for each disease are unique. Thus, common mediating pathways across all three networks are unlikely to have arisen from shared shortest paths between identical sets of genes.

Both the Gene Ontology and the KEGG gene set collections were downloaded from the Molecular Signature DataBase (MSigDB)^48^ on April 3, 2018 (http://software.broadinstitute.org/gsea/msigdb). This GO gene set collection includes 4,436 Biological Process (BP) terms and 15,578 genes, and the KEGG collection includes 186 pathways and 5,266 genes.

### Assessing significance by matching degree distribution of random samples through binning

To compute p_cent_, we sort nodes by degree and place nodes of increasing degree into one bin, with all nodes of the same degree placed in the same bin, until the size of the bin is above a threshold. The algorithm also checks the last bin, and merges it with the previous bin if the size of the last bin is less than half of the size threshold. We used a bin size of *b* = 20 for the results presented here, as this appeared to give reasonable sized bins and to avoid combining nodes with too large a degree range. Supplementary Figure S3 shows the bin sizes and degree ranges for *b* = 20.

To determine the algorithm’s sensitivity to this bin size, we compared p_cent_ values calculated using bin size *b* = 20 to p_cent_ values calculated using size thresholds of 10 and 40 (i.e., *b*/2 and 2*b*). Table 5 shows that p_cent_ values calculated using different bin size thresholds are highly correlated. Our conclusion is that the p_cent_ values are fairly robust to two-fold variation in the bin size, suggesting that our somewhat arbitrary choice of size 20 has at most a modest impact on the results.

**Table 5:** Effect of bin sizes in random sampling on p_cent_ calculation. The table shows the Pearson correlations between the p_cent_ values calculated using pairs of bin sizes (*b*) from the set {10,20,40}. The p_cent_ values are highly correlated between different bin sizes (Python Scipy p-values for the correlations are below 0.0001 for all pairs).

Pearson Correlation				
		Asthma	BPD	COPD
GO BP	p_cent_(*b* = 10) vs. p_cent_(*b* = 20)	0.9840	0.9664	0.9652
	p_cent_(*b* = 20) vs. p_cent_(*b* = 40)	0.9903	0.9848	0.9620
KEGG	p_cent_(*b* = 10) vs. p_cent_(*b* = 20)	0.9902	0.9982	0.9806
	p_cent_(*b* = 20) vs. p_cent_(*b* = 40)	0.9793	0.9762	0.9982

The process to compute *p*_med_ is identical to that for computing *p*_cent_, except for the bin sizes. Here, we chose 5 for the bin size threshold, as it generated a reasonable range of bin sizes. As above, the resulting p_med_ values were analogously determined to be robust to the choice of this size threshold; Pearson correlations between p_med_ values calculated using bin sizes 3, 5, and 10 range from 0.9960 to 0.9998, all with Python Scipy p-values for the correlation <0.0001.

## Supplementary information

Supplemental Figures

Full experimental results

Novel disease-mediating pathways

## Data Availability

All programs we designed and implemented for this study are available for download at https://github.com/TuftsBCB/pathway-centrality. Data generated during our experiments are in supplementary information files, and also accessible at http://bcb.cs.tufts.edu/jpark/pathway-centrality/. This repository includes an excel file containing our full experimental results, two pdf files containing two Venn diagrams and twelve R plots, and three cytoscape session files.
