# Supplementary material for: Pathway centrality in protein interaction networks identifies putative functional mediating pathways in pulmonary disease
Source: Sci Rep. 2019 Apr 10;9:5863. doi: 10.1038/s41598-019-42299-3 (PMC6458310; doi:10.1038/s41598-019-42299-3)
Supplement: Supplementary file 1 — Supplemental Figures [file 41598_2019_42299_MOESM1_ESM.pdf]

Pathway centrality in protein interaction networks identifies putative functional mediating pathways in pulmonary disease

Jisoo Park[1,*], Benjamin J. Hescott[2] , and Donna K. Slonim[3,4,+]

[1] School of Medicine, University of California, San Diego, CA 92093, USA
[2] College of Computer and Information Science, Northeastern University, Boston, MA 02115, USA
[3] Department of Computer Science, Tufts University, Medford, MA 02155, USA
[4] Department of Immunology, Tufts University School of Medicine, Boston, MA 02111, USA

Corresponding authors: *jisoo.park@tufts.edu +slonim@cs.tufts.edu

Supplemental Figures

Figure S1.
R-plots showing that pathways with $p_{cent} < 0.05$ are likely to have low p_{med}. The plots comparable to that in Figure 2b for all combinations of disease and pathway collections are included here. Black bars highlight the pathways with $p_{cent}<0.05$

Figure S2.
Venn diagrams for disease gene sets and differentially expressed genes.
a. Overlap between disease genes for three pulmonary diseases.
b. Overlap between differentially expressed genes in three pulmonary diseases.

Figure S3.
Number of genes in buckets generated using a size threshold of 20 for random sampling in p_{cent} calculation. All nodes are sorted by degree and placed into one bucket, until the size of bucket is greater than 20. The size of buckets is not always 20, as all same-degree nodes are placed in the same bucket.

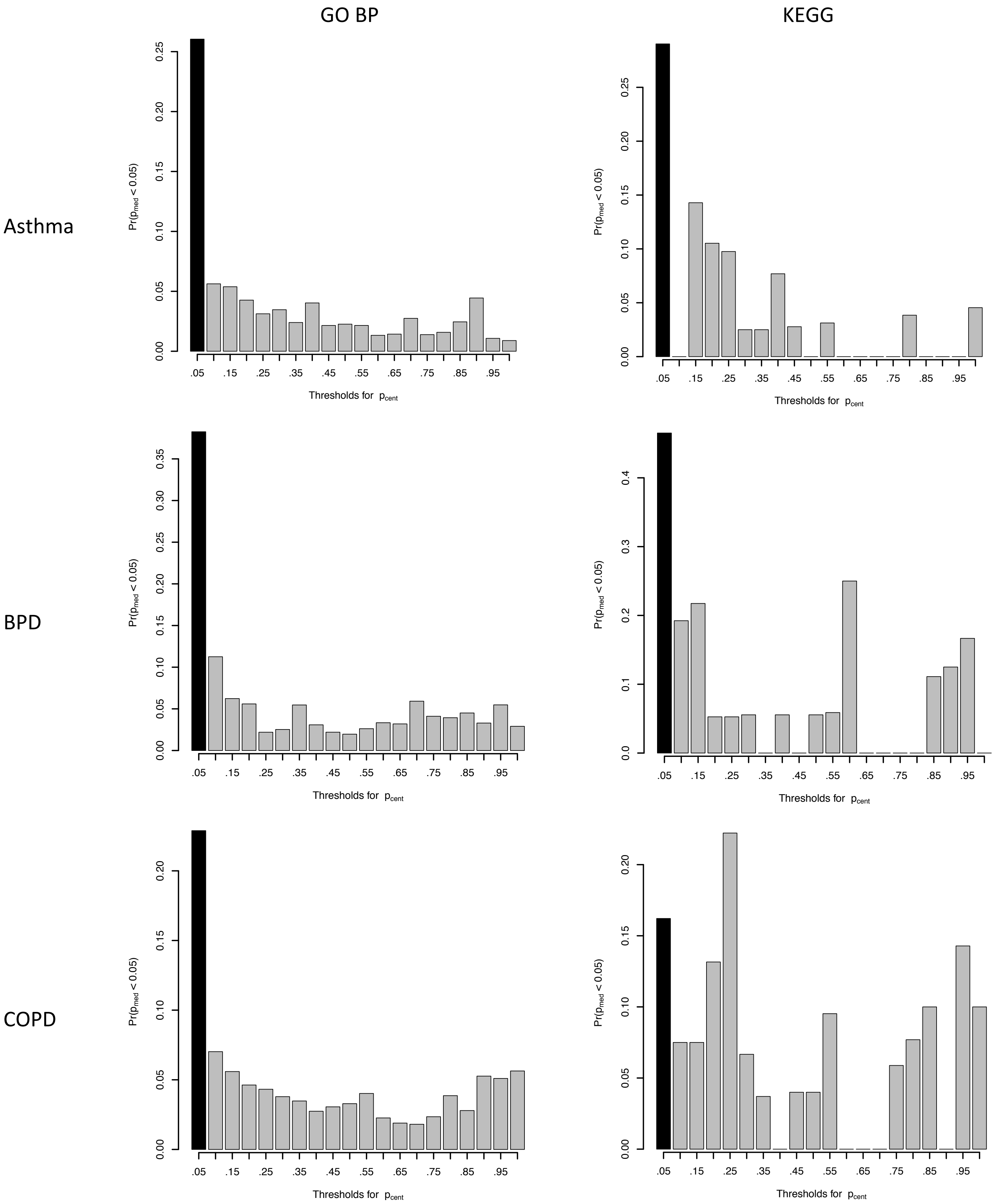

Figure S1.

R-plots showing that pathways with $p_{cent} < 0.05$ are likely to have low p_{med}. The plots comparable to that in Figure 2b for all combinations of disease and pathway collections are included here. Black bars highlight the pathways with $p_{cent}<0.05$

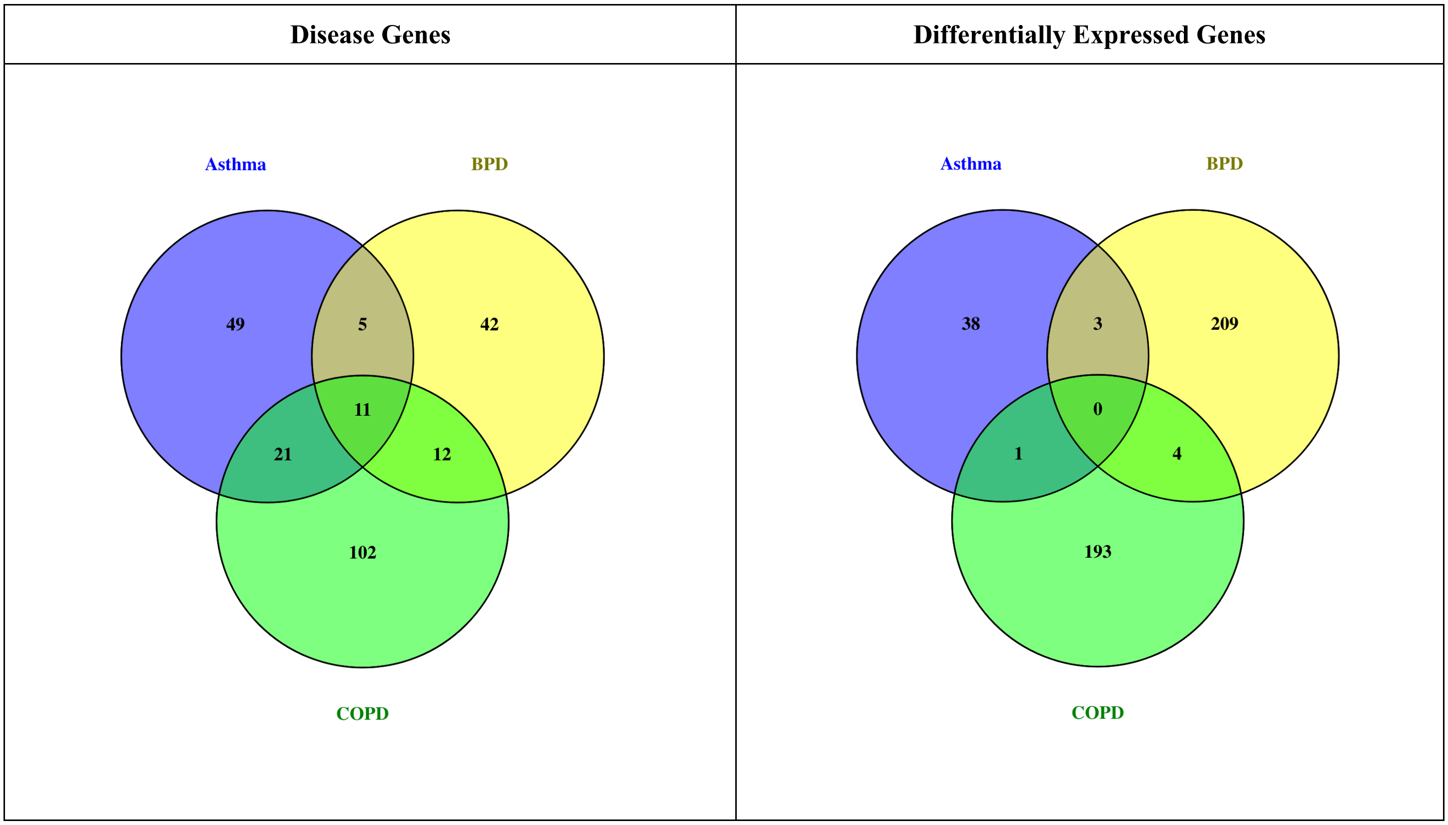

Figure S2.

Venn diagrams for disease gene sets and differentially expressed genes.

a. Overlap between disease genes for three pulmonary diseases.

b. Overlap between differentially expressed genes in three pulmonary diseases.

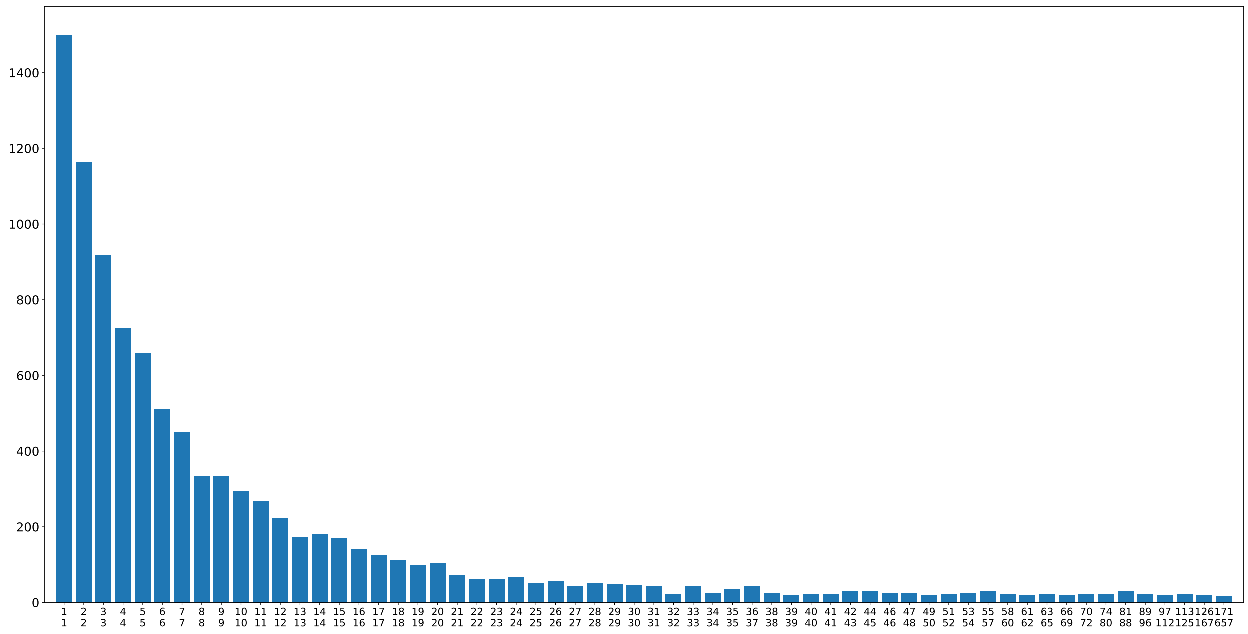

Figure S3.

Number of genes in buckets generated using a size threshold of 20 for random sampling in p_{cent} calculation. All nodes are sorted by degree and placed into one bucket, until the size of bucket is greater than 20. The size of buckets is not always 20, as all same-degree nodes are placed in the same bucket.
